# A Model for the Application of Target-Controlled Intravenous Infusion for a Prolonged Immersive DMT Psychedelic Experience

**DOI:** 10.3389/fphar.2016.00211

**Published:** 2016-07-14

**Authors:** Andrew R. Gallimore, Rick J. Strassman

**Affiliations:** ^1^Computational Neuroscience Unit, Okinawa Institute of Science and Technology Graduate UniversityOkinawa, Japan; ^2^Department of Psychiatry, University of New Mexico School of MedicineAlbuquerque, NM, USA

**Keywords:** psychedelic drugs, dimethyltryptamine, ayahuasca, hallucinations, consciousness, intravenous infusion, pharmacokinetic modeling

## Abstract

The state of consciousness induced by *N,N*-dimethyltryptamine (DMT) is one of the most extraordinary of any naturally-occurring psychedelic substance. Users consistently report the complete replacement of normal subjective experience with a novel “alternate universe,” often densely populated with a variety of strange objects and other highly complex visual content, including what appear to be sentient “beings.” The phenomenology of the DMT state is of great interest to psychology and calls for rigorous academic enquiry. The extremely short duration of DMT effects—less than 20 min—militates against single dose administration as the ideal model for such enquiry. Using pharmacokinetic modeling and DMT blood sampling data, we demonstrate that the unique pharmacological characteristics of DMT, which also include a rapid onset and lack of acute tolerance to its subjective effects, make it amenable to administration by target-controlled intravenous infusion. This is a technology developed to maintain a stable brain concentration of anesthetic drugs during surgery. Simulations of our model demonstrate that this approach will allow research subjects to be induced into a stable and prolonged DMT experience, making it possible to carefully observe its psychological contents, and provide more extensive accounts for subsequent analyses. This model would also be valuable in performing functional neuroimaging, where subjects are required to remain under the influence of the drug for extended periods. Finally, target-controlled intravenous infusion of DMT may aid the development of unique psychotherapeutic applications of this psychedelic agent.

## Introduction

*N,N*-dimethyltryptamine (DMT) produces some of the most extraordinary changes in consciousness of any naturally-occurring psychedelic substance. Users consistently report the complete replacement of awareness of the normal waking world with a bizarre and complex “alternate universe” filled with a variety of visual objects, including what appear to be sentient, intelligent, and powerful “beings,” many of which actively interact with the individual (Strassman, 2001, 2008; Luke, 2011; Gallimore, 2013). Furthermore, the endogenous production of DMT in humans is well-established (Barker et al., 2012), although the biological significance of this remains to be elucidated. DMT is actively transported across the blood brain barrier in rats and dogs (Sangiah et al., 1979; Takahashi et al., 1985; Yanai et al., 1986), and a similar mechanism plausibly exists in humans. DMT is also a substrate for the human serotonin and monoamine vesicular transporters (Cozzi et al., 2009). The key enzyme for its production, indolethylamine N-methyltransferase, has been detected in the brain, pineal gland, and retina of primates (Cozzi et al., 2011). Taken together, these data suggest that DMT may have a significant role in human neurophysiology, consciousness, and the visual system.

Clinical psychedelic drug research has resumed after a generation's hiatus, and its scope is expanding rapidly. Modern functional neuroimaging techniques are revealing the neural accompaniment of these altered states of consciousness (Vollenweider et al., 1997; Carhart-Harris et al., 2012; Roseman et al., 2014; Tagliazucchi et al., 2014; Gallimore, 2015; Nichols, 2016). However, comparable thoroughgoing analyses of their phenomenology are lacking. This is surprising because of how unusual and highly replicable are the subjective effects of the psychedelic drugs in general, in particular those of DMT. The profound and easily reproducible effects of DMT and other psychedelics may provide valuable insights into the structure of the human mind—the central focus of psychology itself. As such, in addition to their neural correlates, the phenomenology and content of the DMT state calls for rigorous academic enquiry.

Studies of DMT in humans began in the 1950s (Boszormenyi and Szara, 1958; Sai-Halasz et al., 1958; Szara, 2007; Gallimore and Luke, 2015), when its mind-altering effects were explored as a form of chemically-induced psychosis. However, such studies simply classified the complex visual effects as “hallucinations” with no further analysis. Neither do most modern studies purporting to examine the psychological effects of psychedelic drugs routinely provide detailed descriptions of the altered state (Riba et al., 2001; Gouzoulis-Mayfrank et al., 2005). An exception is the largest clinical study of DMT to date, which paid careful attention to the content of the DMT experiences of nearly five dozen volunteers administered a wide range of DMT doses (Strassman et al., 1994; Strassman, 1995, 2001).

The time course of DMT administered via inhalation of vaporized freebase or intravenous injection of a water-soluble salt is brief. The onset is rapid and overwhelming, with full effects noted within 2 min of administration. Subjective effects are usually fully resolved within 20–30 min. A powerful “rush” heralds the effect of a fully psychedelic dose of DMT, marked by a sense of tremendous acceleration and psychic and somatic tension. These culminate in the dissociation of consciousness from bodily awareness and entry into a “world of light” characterized by extremely complex visions. Users frequently report the sense of receiving “information,” as well as the conviction that what is being observed feels as if it were an autonomous alternate world rather than a dream or hallucination. A commonly heard refrain is that the experience was “more real than real.”

The inability to induce psychological tolerance to repeated full-psychedelic doses of intravenous DMT administered by bolus injection (Strassman et al., 1996) makes it unique among classic serotonergic psychedelics: e.g., LSD (Belleville et al., 1956). This property renders DMT amenable to administration by continuous intravenous infusion, in which the drug is administered at a predetermined rate over a period of time and subjective effects can be prolonged.

Gouzoulis-Mayfrank et al. (2005) used such an approach to study the psychological effects of both DMT and ketamine. This group's infusion rates were established by observing the subjective effects of the drug, rather than taking into account its underlying pharmacokinetics or pharmacodynamics. That three of the 15 subjects dropped out of the study because of adverse psychological reactions suggests that this model resulted in an overly high infusion rate. A more pharmacokinetically-informed approach to infusion will allow the attainment and maintenance of a stable blood—and presumably brain—concentration of DMT, and thus provide a safe and effective prolonged immersion in the unique DMT state.

## Methods and results

Target-controlled intravenous infusion is a methodology developed for use in general anesthesia, during which it is essential that the concentration of drug at the target site (the brain) be established and stably maintained (Kenny and White, 1990; Absalom et al., 2016). If drug levels drop too low, the patient may rouse during the procedure, and rising brain levels may result in potentially life-threatening effects. Computer-assisted infusion devices are now routinely used to ensure anesthetic levels remain within the required therapeutic window. The foundation of these systems is a mathematical model of the pharmacokinetics and pharmacodynamics of the anesthetic drug (Gambus and Troconiz, 2015). For a drug to be suitable for target-controlled infusion it should ideally possess a number of characteristics listed in Table 1 (Miller, 1994). Fortunately, the water-soluble DMT salt used for intravenous administration meets all of these criteria.

**Table 1:** **Desired characteristics of drugs for continuous IV infusion (Miller, 1994)**.

1. Water-soluble
2. Rapid onset of action
3. Short duration of effect
4. Lack of tolerance
5. High clearance rate with minimal tendency for accumulation
6. No active metabolites
7. High therapeutic index
8. Minimal side-effects

**Figure 1:**
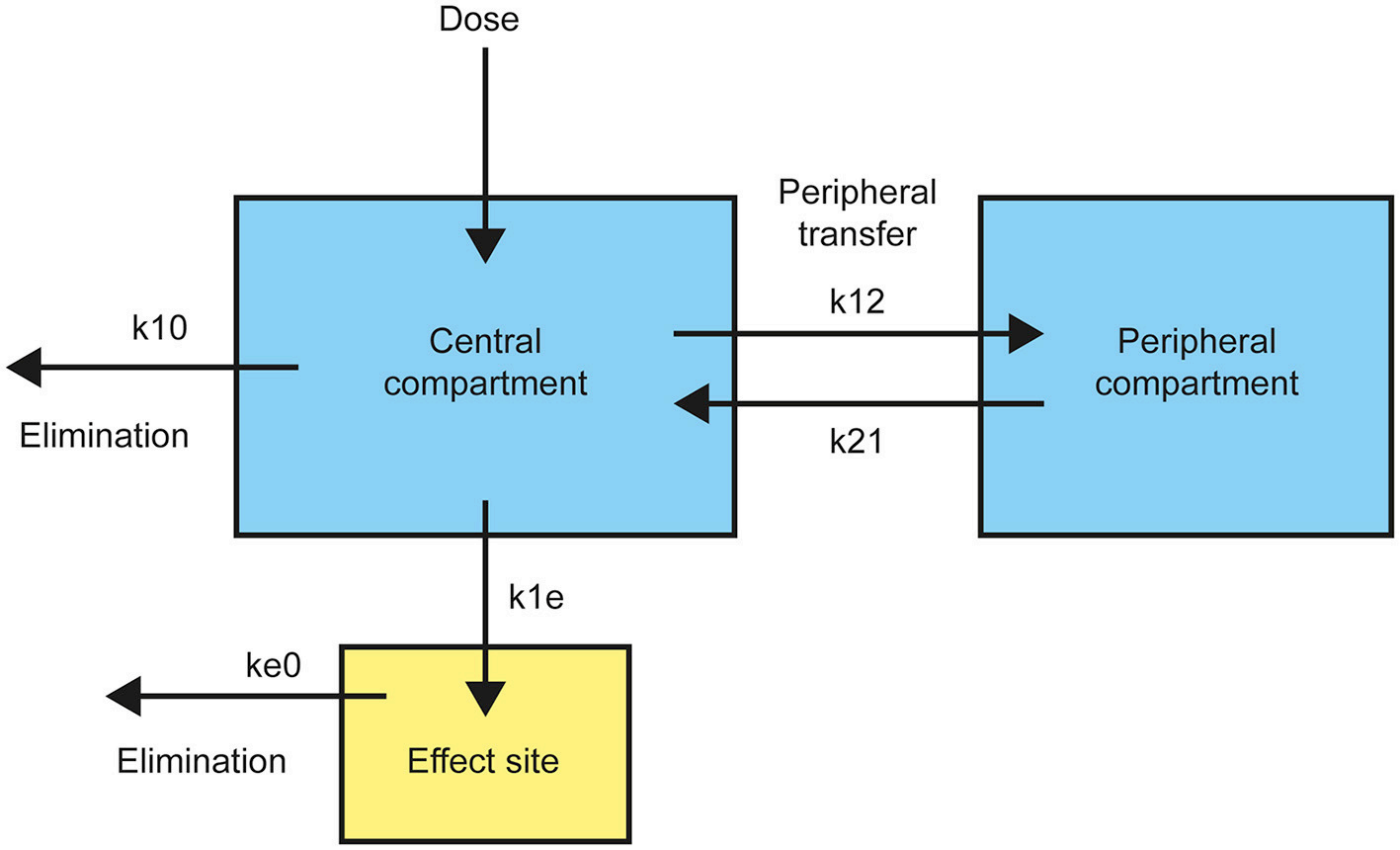
**Structure of a standard two-compartment (plus effect site) pharmacokinetic model with transfer and elimination rates**.

As a drug is introduced into the body by intravenous injection, it is rapidly diluted and distributed by the blood. It also equilibrates to various degrees with peripheral tissues—dependent on specific drug properties and degree of vascularization of the relevant tissues—and with the effect site itself. The elimination of the drug from the body also begins immediately, by a combination of enzymatic transformation, and urinary and/or biliary excretion. Pharmacokinetic models must take into account both intrinsic and drug-specific factors that affect drug absorption, distribution, and elimination. Single-compartment models only consider the rapid dilution of the drug in the main vascular compartment, whereas two- and three- compartment models also take into account equilibration of the drug with peripheral tissues. The pharmacokinetics of most anesthetics are best fitted to either a two- or three-compartment model (Shafer and Gregg, 1992). Since DMT turns out to be best fitted to a two-compartment model (see later), only this type will be described here (Figure 1).

Drug is introduced into the central compartment to rapidly achieve a plasma concentration, *C*_*T*_, dependent on the volume of distribution, *V*_*C*_. The drug is removed from the central compartment, quantified by a drop in plasma concentration over time, by both elimination and equilibration with the peripheral compartment. This rate of plasma concentration decline is controlled by the elimination rate constant, *k*_10_, and the relative rates of movement from the central to the peripheral compartment, *k*_12_, and in the opposite direction, *k*_21_. The overall rate, *R*_*T*_, obeys the differential equation:

$$\begin{array}{l} R_{T}=C_{T}\times V_{C}\left(k_{10}+\;k_{12}e^{{-k}_{21}t}\right) \end{array}$$

Complete equilibration with the peripheral compartment is reflected in the exponential term decaying to zero and the steady state elimination rate, *R*_*SS*_, is:

$$\begin{array}{l} R_{SS}=C_{T}\times V_{C}k_{10} \end{array}$$

Transfer from the central compartment to the effect site, *R*_1__*E*_, is generally modeled as a first-order process with rate constant, *k*_1__*e*_:

$$\begin{array}{l} R_{1E}=\;C_{T}k_{1e} \end{array}$$

To maintain a constant plasma, and thus effect site, concentration, the infusion rate must equal the overall removal rate, *R*_*T*_. Since this is not constant, except at steady state, the infusion rate must be adjusted to approximate the decline in *R*_*T*_ over time. This requires the determination of the pharmacokinetic parameters: *V*_*C*_, *k*_10_, *k*_12_, and *k*_21_, which can be achieved by fitting time-series blood sampling data to a pharmacokinetic model.

To establish that the pharmacokinetics of DMT make it suitable for target-controlled infusion, we used DMT plasma concentration data from a previous study (Strassman and Qualls, 1994). The details are provided in the original paper. Briefly, each subject receiving a “fully psychedelic” dose of DMT was administered either 0.2 or 0.4 mg/kg DMT fumarate over 30 s through an indwelling forearm intravenous catheter, followed by a 15 s flush with sterile saline. Blood samples were drawn before the infusion and at 2, 5, 10, 15, 30, and 60 min from the end of the infusion (45 s after the infusion began). Plasma DMT concentration in each sample was then determined using gas chromatography-mass spectrometry (GC-MS). A total of nine subjects were used in the analysis (9 sets each of 0.4 and 0.2 mg/kg time series data).

These time-series data were fitted to one-, two-, and three-compartment pharmacokinetic models. The naïve averaged data approach is the most straightforward technique for fitting pharmacokinetic data, in which plasma concentration at each time point is averaged over all subjects to generate a mean dose-response curve. This curve is then fitted to a pharmacokinetic model. However, methods taking into account both fixed and random effects on the dose-response—for example, non-linear mixed effects modeling (NON-MEM)—often give more reliable parameter estimates. These methods also allow identification of parameter covariates, such as weight or age (Mould and Upton, 2012).

**Figure 2:**
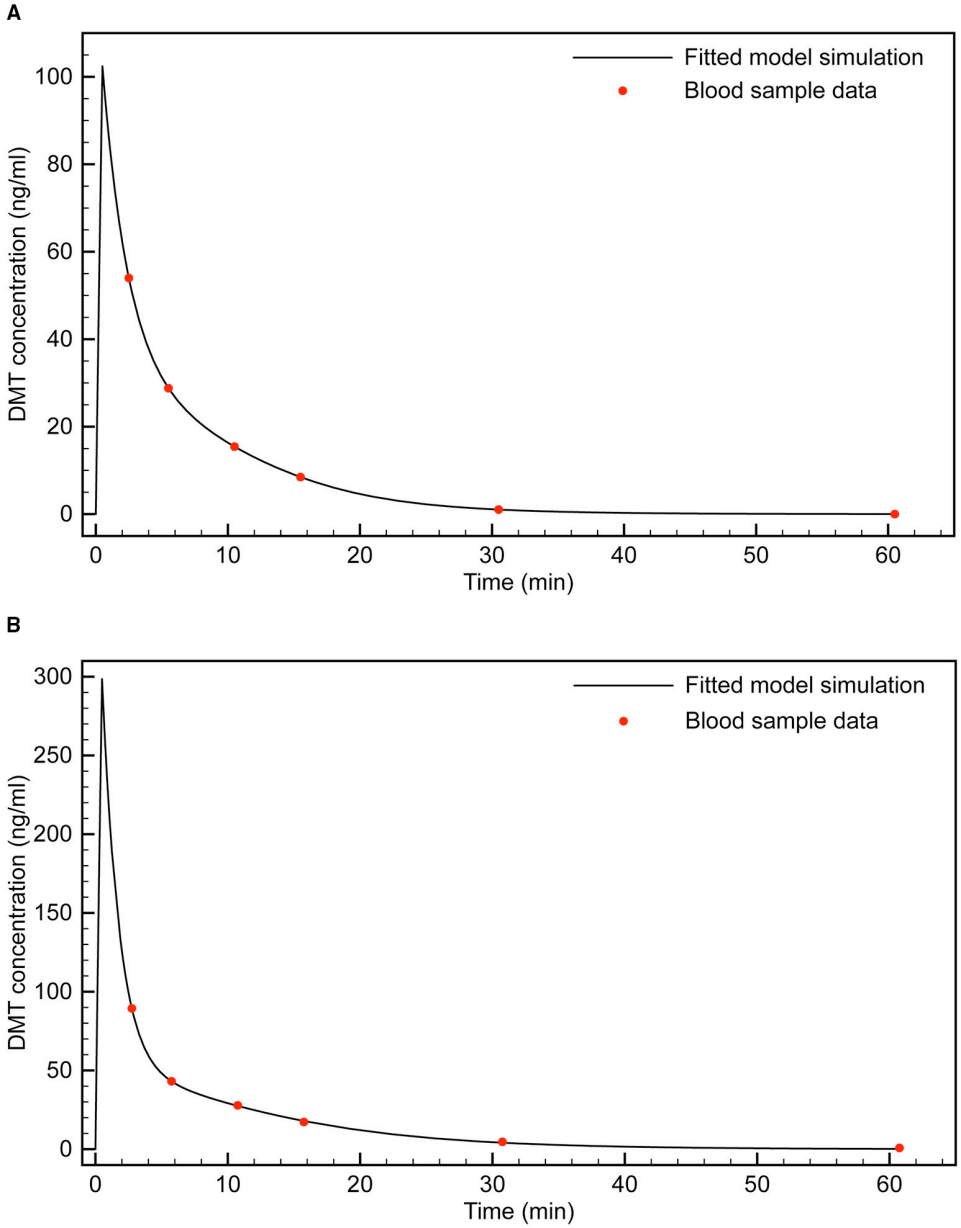
**Fitting of two-compartment model with enzymatic clearance to blood sample data**. **(A)** 0.4 mg/kg bolus; **(B)** 0.2 mg/kg bolus.

Using the Matlab Simbiology toolkit (Mathworks, Inc.), we fitted the 0.2 and 0.4 mg/kg time- series data separately. Both datasets were best fitted to a two-compartment model with enzymatic clearance, consistent with Michaelis-Menten kinetics (Figure 2). This comports with the well-established rapid metabolism of DMT by monoamine oxidase (MAO) A (Barker et al., 1980; Sitaram et al., 1987; Riba et al., 2015). Table 2 shows the population parameter estimates obtained.

**Table 2:** **Fitted pharmacokinetic parameters for 2-compartment model with enzymatic clearance**.

	***V_c_* (L)**	***V_p_* (L)**	***K_m_* (mg/L)**	***V_m_* (mg/min)**	***Q*_12_ (L/min)**
0.4 mg/kg	0.093	0.34	21.48	2.00	0.056
0.2 mg/kg	0.14	0.23	9.52	1.07	0.046
*k*_12_ = *Q*_12_/ *V*_c_; *k*_21_ = *Q*_12_/*V*_p_					

These parameter estimates are for an “average” individual (i.e., obtained from the mean plasma concentration across the nine subjects at each time point), although the dose-concentration response varied considerably among subjects. The mean coefficients of variation in plasma DMT concentration, between 2 and 30 min post-infusion, across the 0.2 and 0.4 mg/kg groups were 83 and 58%, respectively. There was also variation in the estimated parameters depending on the dose. Since 0.4 mg/kg was the most reliable dose for inducing the fully psychedelic DMT experience, we sought an infusion model that reaches and maintains the effect site concentrations observed with this dose. Therefore, the 0.4 mg/kg parameter set was chosen for development of the infusion model. However, comparable results were obtained using the alternative parameter set.

Having established the model parameters, we then sought to extend the model to include the effect site (brain) concentration. The model was simulated using an infusion protocol employed in the original study (i.e., 0.4 mg/kg infusion over 30 s). While subjects were unable to communicate during the peak DMT effects, a number of observations indicated that these peak effects in each subject occurred at approximately 3 min from the beginning of the infusion. First, the lower doses of drug (0.05 and 0.1 mg/kg) were not incapacitating, and provided the opportunity for volunteers to describe the onset, peak, and dissipation of drug effects in a running commentary narrative as they were occurring. With these lower doses, effects peaked at approximately 3 min, began dissipating quickly, and were resolved by 15–20 min. That the time course of peak DMT blood levels is identical across the spectrum of doses suggests that the correspondence between the time course of peak DMT effects and peak plasma levels at higher incapacitating doses also holds true. Acute autonomic responses to DMT also reached their highest levels between 2 and 5 min from the end of the infusion, usually at the former time point. These included pupil diameter, heart rate, and mean arterial blood pressure.

**Figure 3:**
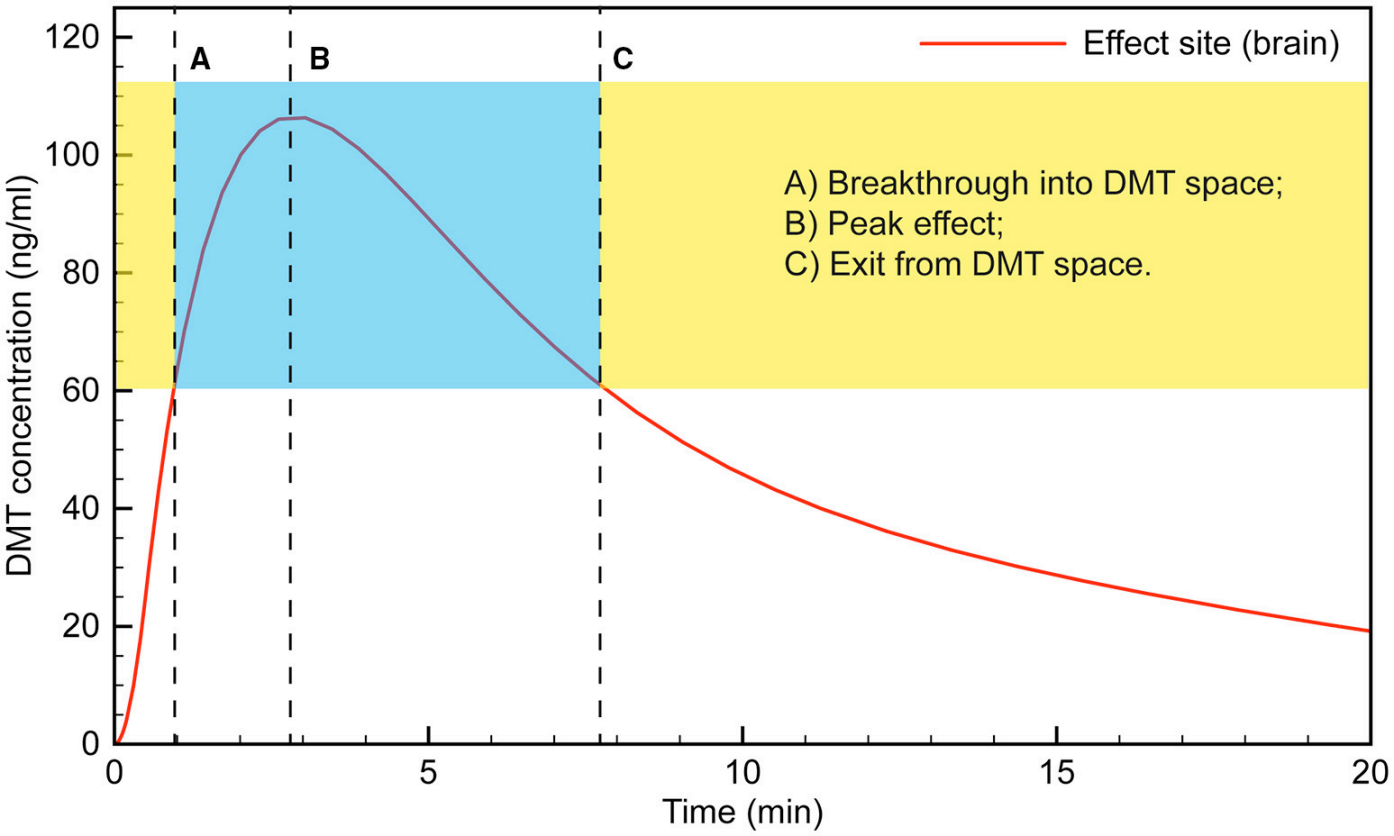
**Simulated time course of DMT brain concentration following a 0.4 mg/kg bolus**.

At the peak effect time, the drug concentration in the central compartment is equal to the concentration at the effect site. This makes it straightforward to model the effect site concentration using the first-order rate equations:

$$\begin{array}{l} R_{1E}=\;C_{T}k_{1e}\\ R_{E0}=\;C_{T}k_{e0} \end{array}$$

where *R*_1*E*_ is the rate of transfer of the drug from the central compartment to the effect site and *R*_*E*0_ is the elimination rate from the effect site, controlled by the rate constants *k*_1*e*_ and *k*_*e*0_, respectively. The parameters were tuned such that the peak effect site concentration was reached at ~3 min. The resulting simulation is plotted in Figure 3 and seems to fit the observations well.

Typically, the subject would appear to transition into full dissociation from the external world toward the end of the 15 s saline flush following the 30 s DMT infusion. This suggests that “breakthrough” into the DMT space occurs when the effect site concentration reaches ~60 ng/ml. The subject then remains in the “space” for the next 6–7 min, with the peak effect occurring at 3 min, when the effect site concentration is just over 100 ng/ml.

Clinical observation of the subjects appears to match this time course, with their maximum level of absorption in the subjective experience occurring at 4–5 min, likely as the initial rush and disorientation began to subside. For example, subjects demonstrated seemingly involuntary deep rhythmic breathing, the mouth being open in a passive regressed manner, and REM-like eye movements behind closed lids. Conversely, subjects appeared to be less under the influence of a high dose of DMT as early as 5 min; e.g., moving their fingers, licking their lips or yawning, taking a deep sighing breath, and stretching their feet, suggesting that the acute drug effect was beginning to lighten. Tellingly, almost every volunteer did not remember the 2 min blood pressure measurement, whereas almost all remembered the 5 min measurement, which is when our model suggests the effect site concentration was again approaching ~60 ng/ml, when exit from the full DMT intoxication occurs.

Having simulated the infusion protocol used in the original study, we then used the model to develop an infusion protocol that would bring the effect site concentration smoothly to the predetermined level to ensure “breakthrough” without significant overshoot, and to keep the concentration stable indefinitely. A 100 ng/ml brain concentration was typically achieved during the most intense period of the DMT experience with a 0.4 mg/kg bolus, and this concentration was chosen as the desired target concentration for the infusion. However, lower or higher concentrations can be achieved using an analogous protocol with modified infusion rates.

We developed our infusion model using the bolus-elimination-transfer (B.E.T.) methodology, upon which anesthesia infusion protocols are often based (Coetzee, 2012). An initial bolus, B_0_, is used to rapidly bring the plasma concentration to the desired level, *C*_*T*_:

$$\begin{array}{l} B_{0}=\;C_{T}V_{C} \end{array}$$

The infusion rate is then calculated to equal the sum of the elimination rate, *E*, and the transfer rate from the central to the peripheral compartment, *T*.

$$\begin{array}{l} E=\;V_{C}\times k_{10}C_{T}\\ T=\;V_{C}\times k_{12}e^{{-k}_{21}t}C_{T} \end{array}$$

The sum of *E* and *T* gives the overall infusion rate as defined earlier:

$$\begin{array}{l} R_{T}=C_{T}\times V_{C}\left(k_{10}+\;k_{12}e^{{-k}_{21}t}\right) \end{array}$$

Since the model-fitting established that E is dominated by enzymatic clearance, *k*_10_ must be reformulated in terms of Michaelis-Menten kinetics:

$$\begin{array}{l} k_{10}C_{T}=\;\frac{V_{m}C_{T}}{K_{m}+C_{T}} \end{array}$$

This gives the maintenance infusion rate, *R*_*T*_, as:

$$\begin{array}{l} R_{T}=\;\frac{V_{m}C_{T}}{K_{m}+C_{T}}+\;{C_{T}V}_{C}k_{12}e^{{-k}_{21}t} \end{array}$$

Since the first term in the *R*_*T*_ equation depends only on the plasma concentration, it becomes constant when a steady state concentration is reached. The exponential term is only important before steady state is reached, in the initial stage of the infusion. It then decays to zero. Assuming the steady state concentration is the desired effect site concentration, *C*_*T*_, the maintenance infusion rate, *R*_*ss*_, can be calculated:

$$\begin{array}{l} R_{ss}=\;\frac{V_{m}C_{T}}{K_{m}+C_{T}} \end{array}$$

Using the estimated model parameters, this gives a steady state infusion rate of 0.93 mg/min. However, during the first few min of the infusion, the exponential term is large; i.e., there is rapid transfer of drug from the central to the peripheral compartment. This rate of transfer peaks at 3.3 mg/min, at around 2.3 min, before declining rapidly. The infusion rate must be set to compensate for this transfer, being decreased gradually until *R*_*ss*_ is reached. This variable infusion rate is essential to attaining and maintaining a stable effect site concentration. If the initial rate is too low following the initial bolus, the effect site concentration plummets well below that desired and is not maintained. Conversely, if a high initial infusion rate is maintained, the effect site concentration continues to increase. It is possible that this accounts for the relatively high rate of volunteers dropping out of the Gouzoulis-Mayfrank study (Gouzoulis-Mayfrank et al., 2005).

**Figure 4:**
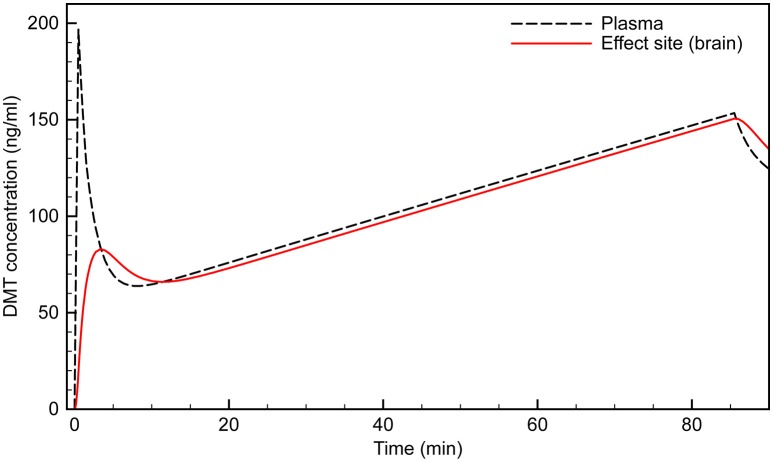
**Simulated time course of plasma and effect site DMT concentration using the (Gouzoulis-Mayfrank et al., 2005) protocol**.

To examine the possibility of effect site concentration overshoot, we performed simulations using the Gouzoulis-Mayfrank infusion protocol: 0.3 mg/kg bolus, followed by an infusion beginning at 1.5 min, at a rate of 0.02 mg/kg/min over 84 min. Figure 4 shows the expected effect site concentration over this infusion period for a 75 kg subject. The initial bolus produces an effect site concentration of 80 ng/ml; i.e., a breakthrough dose. Once the infusion begins, however, the concentration rises steadily, and reaches 150 ng/ml by the end of the session. This is a very high concentration and is certain to produce extremely intense effects in almost all individuals.

**Figure 5:**
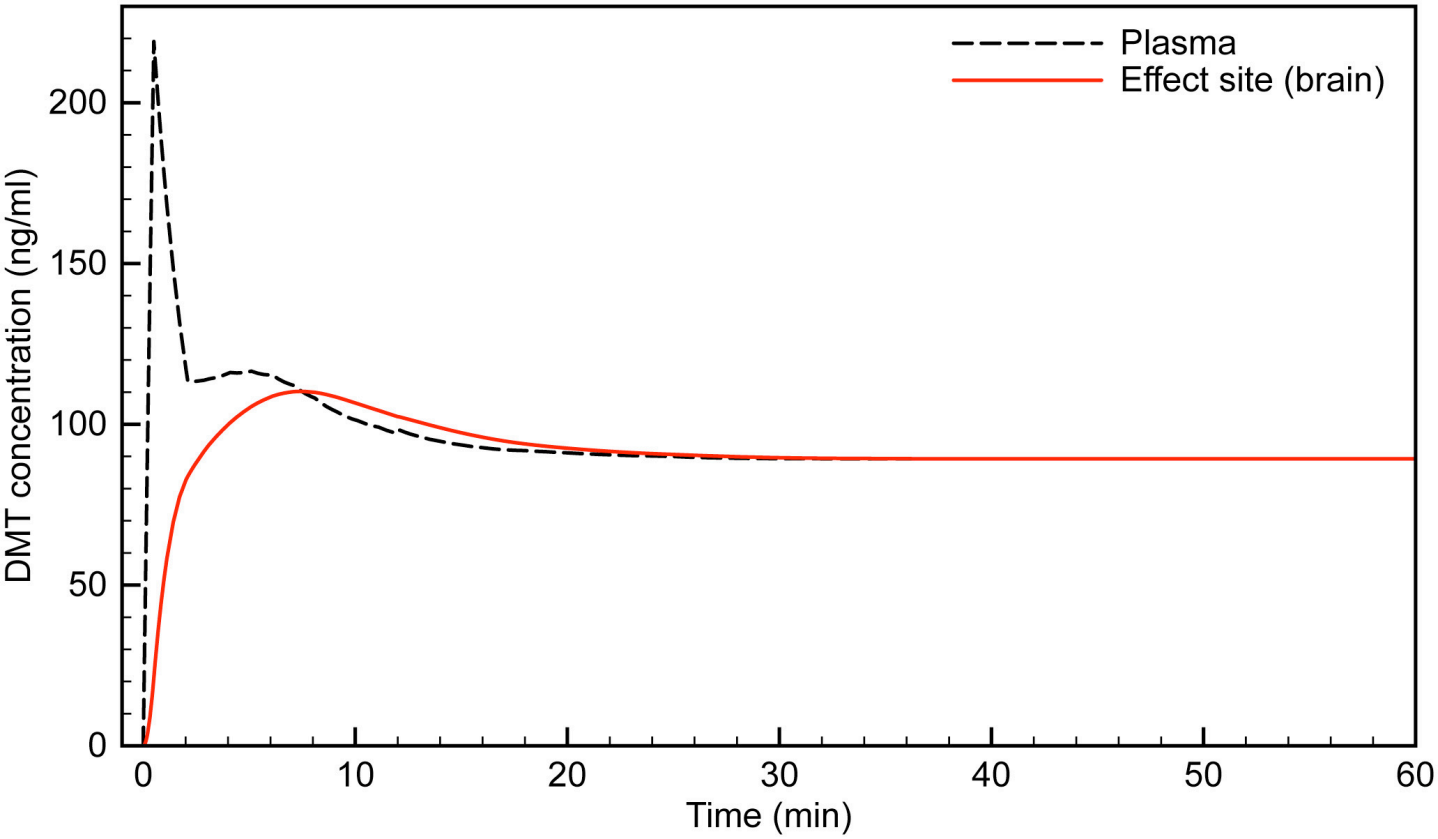
**Simulated time course of infusion protocol designed to reach and maintain effect site concentration of ~100 ng/ml**.

Using our PK model, we developed an infusion protocol that maintains an effect site concentration of ~100 ng/ml in a 75 kg subject (Figure 5). An initial bolus of 25 mg infused over 30 s rapidly brings the effect site concentration to just over 100 ng/ml. Although the plasma concentration spikes at over 200 ng/ml, the desired effect site concentration is reached smoothly with very little overshoot. The infusion begins at 2 min at a rate of 4.2 mg/min. The infusion is updated every min, and decreases according to the peripheral transfer rate decay (the exponential term in the *R*_*T*_ equation). Steady state does not occur until after 20 min of infusion, after which a constant maintenance infusion rate of 0.93 mg/min is employed.

The blood sample data used in this work was from a previous study (Strassman and Qualls, 1994) approved by the Scientific Advisory Committee of the General Clinical Research Center (GCRC) and Human Research Review Committee of the University of New Mexico School of Medicine, Albuquerque, the New Mexico State Pharmacy Board, the US Drug Enforcement Administration, and the US Food and Drug Administration. Witnessed written informed consent was obtained from all subjects, and confidentiality and anonymity were maintained throughout the study.

## Discussion

The phenomenological content of dream states (Schredl and Hofmann, 2003; Kahan and Laberge, 2011; Thomas et al., 2015) and hallucinations in psychotic disorders have been studied extensively (Jardri et al., 2014; Wilkinson, 2014), with the aim of deepening our understanding of the structure of normal and abnormal states of consciousness. However, whilst the endogenous human hallucinogen DMT reliably and reproducibly generates one of the most unusual states of consciousness available, its phenomenology has only begun to be characterized. One of the reasons for this is its short duration of action. A technology for extending DMT experiences in a controlled manner beyond what is achievable using bolus administration therefore would be of great value.

Modern target-controlled infusion protocols employ algorithms that allow the infusion rate to be calculated and adjusted in real time, such that the effect site drug concentration can be raised and lowered in order to control, for example, the level of anesthesia (Bailey and Shafer, 1991; Shafer and Gregg, 1992). Our analysis highlights the potential of using the target-controlled infusion methodology for extended DMT sessions. Using time-series blood sampling data and pharmacokinetic modeling, we propose that the unique pharmacological characteristics of DMT make it suitable for administration by target-controlled intravenous infusion. These characteristics include a rapid and short-acting effect, and lack of acute tolerance to its subjective effects. Such methods could be used to control the depth of the experience during a DMT session, moving the subject into more intense levels of DMT intoxication or lowering them back into more manageable levels to provide both respite and easier communication with the research team.

The methodology developed here is the first step toward a protocol that is ready for use in research subjects. The purpose of this modeling is to provide a proof-of-principle that the pharmacokinetic and pharmacodynamic properties of DMT are such that stable effect site concentrations can be achieved using target-controlled infusion. More extensive sampling and more detailed pharmacokinetic modeling are required to establish definitive population parameters, the extent of inter- and intra-subject variability, and covariates.

As with any drug administered by target-controlled infusion, subject covariates including weight, age, gender, and liver function create significant inter-subject and intra-subject variability in the dose-concentration response (White et al., 2008). In this study, the mean coefficients of variation in plasma DMT concentration across the 0.2 and 0.4 mg/kg groups were 83 and 58%, respectively. Although this variability appears large, dose-response coefficients of variation between 40 and 60% for anesthetic agents (e.g., propofol) administered by bolus injection are typical. However, the response variability for continuous infusion protocols is always lower than that observed for bolus injection (Hu et al., 2005). Even with well-established infusion protocols, plasma drug concentrations typically deviate 20–30% above or below the target concentration (Coetzee et al., 1995). For this reason, most anesthesia infusion protocols undergo a number of iterations as pharmacokinetic models are updated and refined.

Thus, the variability observed in this initial modeling study does not preclude the development of a target-controlled infusion protocol for DMT. In addition, despite the large dose-concentration variability, the variability in the relationship between the dose and the subjective response was much lower. The coefficients of variation of the subjective response scores, as measured by the Hallucinogen Rating Scale, an instrument designed specifically to assess the DMT effect, were 46 and 32% for the 0.2 and 0.4 mg/kg groups, respectively (Strassman et al., 1994). This 45% lower subjective response variability compared to dose-concentration variability suggests that a broader target concentration window exists for attainment of the desired subjective effect than might be predicted from dose-concentration variability alone. Therefore, the target concentration accuracy typically achieved with anesthetic agents is likely to be suitable in a DMT infusion protocol.

The DMT-containing plant-based decoction ayahuasca provides an extended “DMT experience” lasting several hour (Shanon, 2003; Frenopoulo, 2005; Schmidt, 2012). The phenomenology of the ayahuasca state has been the subject of more extensive analyses than that of pure DMT (Shanon, 2005). However, to render the preparation orally-active, the ayahuasca brew must contain both a DMT-containing plant as well as one containing a beta-carboline MAO inhibitor, such as harmaline (McKenna et al., 1984). Since beta-carbolines themselves possess psychoactive properties and may potentiate the effects of other psychoactive alkaloids in the plant mixture (Callaway et al., 1999), the subjective effects of ayahuasca differ from those of pure DMT. Furthermore, it is not possible to precisely regulate and maintain the effect-site DMT concentration with an oral preparation.

There are a number of research questions that a successful application of target-controlled IV infusion of DMT may address. For example, the maximum DMT effect may extend further than what has been previously described in bolus studies. Very high acute doses of DMT typically produce a delirium, and users are unable to recall details of the experience. However, this may result from too rapidly attaining supra-maximal concentrations of drug. Using the method described here, it may be possible to move the individual gradually into a greater level of intoxication while maintaining the characteristic mental clarity associated with fully psychedelic doses.

In addition, this model is applicable to studies of the neural correlates of the psychedelic state as revealed by modern functional neuroimaging techniques, which are also of great interest (Carhart-Harris et al., 2016; Speth et al., 2016). Since such protocols usually require the research subject to be under the influence of the drug for an extended period of time, our methodology would benefit these investigations as well.

Finally, there are potential psychotherapeutic applications. With the resumption of clinical research with psychedelic drugs, projects confirming and extending early research demonstrating the benefit of psychedelic drug-assisted psychotherapy are playing a prominent role. The conditions to which these novel treatments are being applied include depression (Berman et al., 2000; Aan Het Rot et al., 2010; Buchborn et al., 2014), obsessive-compulsive disorder (Moreno and Delgado, 1997), dysphoric psychological accompaniments of terminal illness (Grof et al., 1973; Grob et al., 2011), prisoner recidivism (Hendricks et al., 2014), and substance abuse disorders—including alcohol (Bogenschutz et al., 2015) and tobacco (Mangini, 1998; Krebs and Johansen, 2012). All of the psychedelic substances being used in these studies—LSD, psilocybin, and ayahuasca—exert their effects over the course of 6–12 h, consistent with their pharmacokinetic profiles.

One of the advantages of the rapid onset and short duration of intravenous DMT effects is the ability to enter into and exit out of a highly altered state in short order. The therapeutic potential of this characteristic of DMT was illustrated by an unexpected finding in the DMT tolerance study (Strassman et al., 1996). Normal control volunteers received four fully psychedelic 0.3 mg/kg doses of DMT at 30 min intervals. During the 10–15 min of relative lucidity between doses, they were quite capable of describing both the effects they had just undergone as well as what they anticipated would be the effects of the subsequent dose(s). The overwhelming effects of a psychedelic dose of DMT contributed to a heady and dynamic psychological confluence of self-disclosure, anticipation, anxiety, vulnerability, and intimacy. In addition, we found that nearly every subject's sessions contained an ongoing set of themes, or “storyline.” These themes began with the first dose, evolved over the following two, and resolved and/or culminated in the fourth. The opportunity provided by talking with a therapeutically-trained research team during the inter-bolus periods contributed to the psychological work that these normal volunteers were able to do. We doubt that this would have been possible if the volunteers were under the influence of the drug unabatedly for that same 2.5 h period.

This paradigm of a continuous target-controlled infusion similarly could be turned to therapeutic purposes in a patient population. Using this model, patients could be “titrated” for both the duration and intensity of the DMT state that was most useful for augmenting the psychotherapeutic process. For example, in the case of working through trauma, re-experiencing the feared stimulus in the altered state might be initiated with induction into a mildly altered state of relatively brief duration. In the course of treatment, a more prolonged and intense altered level of consciousness could be applied to a more extensive working through process, broadening and deepening the therapeutic gains begun with shorter and lighter exposures.

In summary, we have described the rationale, and presented the requisite pharmacokinetic calculations, for the development of target-controlled intravenous infusion of the powerful endogenous psychedelic substance, DMT. The successful demonstration of this model would provide a valuable tool in determining the role of DMT in normal and altered states of consciousness, and have broad psycho-heuristic, functional imaging, and clinical applicability.

## Author contributions

AG performed the analyses. RS provided the original blood sample data. AG and RS wrote the paper.

## Funding

This work was funded in part by the Okinawa Institute of Science and Technology Graduate University, Okinawa, Japan.

### Conflict of interest statement

The authors declare that the research was conducted in the absence of any commercial or financial relationships that could be construed as a potential conflict of interest.
